# Bioinformatic approach for the discovery of *cis*-eQTL signals during fruit ripening of a woody species as grape (*Vitis vinifera* L.)

**DOI:** 10.1038/s41598-022-11689-5

**Published:** 2022-05-06

**Authors:** Pedro José Martínez-García, Jorge Mas-Gómez, Jill Wegrzyn, Juan A. Botía

**Affiliations:** 1 Department of Plant Breeding, Centro de Edafología y Biología Aplicada del Segura (CEBAS), CSIC, P.O. Box 164, 30100 Espinardo, Spain; 2 Department of Ecology and Evolutionary Biology, University of Connecticut, Storrs, CT 06269 USA; 3 Department of Neurodegenerative Disease, University College London, London, WC1N 3BG UK; 4 Departamento de Ingeniería de la Información y las Comunicaciones, Universidad de Murcia, 30100 Murcia, Spain

**Keywords:** Gene expression profiling, Transcriptomics, Agricultural genetics, DNA sequencing, Next-generation sequencing

## Abstract

Expression quantitative trait loci (eQTLs) are associations between genetic variants, such as Single Nucleotide Polymorphisms (SNPs), and gene expression. eQTLs are an important tool to understand the genetic variance of gene expression of complex phenotypes. eQTLs analyses are common in biomedical models but are scarce in woody crop species such as fruit trees or grapes. In this study, a comprehensive bioinformatic analysis was conducted leveraging with expression data from two different growth stages, around ripening onset, of 10 genotypes of grape (*Vitis vinifera* L.). A total of 2170 *cis*-eQTL were identified in 212 gene modulated at ripening onset. The 48% of these DEGs have a known function. Among the annotated protein-coding genes, terpene synthase, auxin-regulatory factors, GRFS, ANK_REP_REGION domain-containing protein, Kinesin motor domain-containing protein and flavonol synthase were noted. This new inventory of *cis-*eQTLs influencing gene expression during fruit ripening will be an important resource to examine variation for this trait and will help to elucidate the complex genetic architecture underlying this process in grape.

## Introduction

Many, if not most, of important characters to animal, plant and human research, such as morphological, life history, behavioral traits, as well as many human diseases such as cancer and diabetes are genetically complex. Complex traits are controlled by many genes (polygenic) and/or environmental conditions, as well as encompass phenotypes that are only expressed when the effects of many genes and/or environmental conditions reach a minimum threshold. Understanding the mechanisms behind the differences in complex traits among individuals, populations, and species has been an essential challenge to evolutionary biology since Darwin and Galton^1^. Understanding the phenotypic differences among individuals requires an understanding of the causes of genetic variation in complex traits.

The systematic dissection of the genetic basis of variation in complex traits has become achievable in the last 10 years^2^. Since 2007, genome-wide association studies (GWAS) have detected associations between common genetic variation at thousands of loci for important human diseases^3^. As of June 8, 2021, the catalog of published GWAS (http://www.genome.gov/gwastudies) includes 5106 publications and 161,014 SNPs. Surprisingly, the number of specific mutations identified and demonstrated to be causative has been very low in comparison with the high numbers of genomic loci underlying complex traits. There are two main reasons for the difficulty of understanding GWAS associations. First, that nearby genetic variants are likely inherited together due to co-segregation during meiotic recombination, a phenomenon named linkage disequilibrium (LD), hindering the identification of the causal variants propping the association. Secondly, it is unknown which cell types are causal to the disease as the pathophysiology of complex diseases often involves interactions of many cell types^4^. More interestingly, nearly 90% of these trait/disease associated SNPs (TASs) were located in non-protein-coding regions, suggesting a possible role in gene regulation of these associated variants^5^. Taken together in spite of this amount of GWAS, the amount of reports that have explored the underlying mechanisms of the detected associations is orders of magnitude fewer^6,7^.

One of the biggest challenges in the post-GWAS era lies in connecting additional molecular data with these GWAS findings to functionally illustrate the relationships. The combination of current bioinformatic, statistical, and empirical bench-based methods allows for the downstream elucidation of GWAS-identified trait loci. In this sense, genomics is a very powerful tool to identify expression quantitative trait loci (eQTLs), meaning the genomic loci that control gene-expression differences. The aim of eQTLs is to improve our understanding of the genetic architecture of disease susceptibility and complex traits and to improve our knowledge of how gene networks are organised within an organism^8^. Almost 50% of trait-associated SNPs have a *cis*-acting effect on gene expression^9,10^. According to this, *cis*-eQTL associations have been performed to detect the novel, but weak, associations that do not reach the genome-wide significance level in GWAS without the requirement of increasing the size of the sample^11,12^. Other significant benefits of the detection of eQTLs is that they can provide knowledge about the underlying pathways and the trait mechanism^13^. To characterize functional genetic variation in humans, a large consortium has allowed the development of the Genotype-Tissue Expression (GTEx) project supported by the NIH Common Fund^14^. This resource allows researchers to study the connection between gene expression and genetic variation in a variety of tissues. Studying large amount of tissues or cell types is expensive, laborious, limited to humans and unaffordable for plant species such as woody crops. However, eQTL studies are gaining popularity in plant genetics mainly due to the fact that they represent a efficient approach to short out the tedious procedure of positional cloning, especially for genes underlying quantitative characters^15^. At the same time, eQTL studies of tree species is a real challenge because of sampling methods^16^ since they do not grow easily under controlled environments due to large space requirements. Indeed, few eQTL studies have been conducted in woody species^17–23^. Some of these studies have been carried out in grapes (*Vitis vinifera* L), the target organism of this study. Grapes are one of the first domesticated fruit crops and are one of the most profitable horticultural crops, with around 8 million hectares (ha) of vineyard in the world, the majority destined to produce wine. The development of the grape berry follows a double sigmoid pattern of growth, with véraison marking the beginning of the second growth phase, called ripening^24^. This phase is characterized by some of the most noticeable changes in the grapes: pronounced berry growth, sugar accumulation, decrease of acidity/raise of pH and accumulation/changes in phenolic compounds and aromatics^25,26^. From a physiological point of view, ripening concerns biochemical changes in the pericarp of the fruit and starts when seeds have completed their development and enter dormancy. In the published results by Massonnet et al.^27^, the authors found that in general a high increase of differentially expressed genes (DEGs) was characterized in the majority of varieties between pre-véraison (PV) stage and end of véraison (EV) stage, with a notable decline in the number of DEGs from end of véraison (EV) to harvest (H).

Only a few eQTL studies in grapes have been carried out which study a group of genes of the same pathway^18,21^ and in general studies that surveyed genome-wide expression to study fruit ripening in grapes are scarce. Interestingly, from the 21 eQTLs identified by Huang et al.^18^, 17 were novel loci that do not correspond with known *cis*-regulatory sequences or candidate transcription factors. These results point out the importance of developing studies that survey genome-wide expression to identify an extended inventory of *cis*-regulatory sequences associated with grape ripening.

In this sense, the aim of this study is to integrate the gene expression information with genome structural features during ripening *Vitis vinifera* L. fruit. For that public DNA and RNA data from 10 grape cultivars, 5 red and 5 white cultivars, were used. The integration of both layers of information, structural variants and differential expression, around ripening onset allowed a *cis*-eQTL analysis to identify genes and relevant mechanisms for this complex process. The final inventory of *cis*-eQTLs will be an important resource for future research to understand the mechanism for variation in gene regulation during ripening in this species, and could be considered general markers of ripening in grapes.

## Results

The DNA and RNA data information and also trimming and alignment statistics for each replicate used in this study can be found in Supplemental File 1 Tables S1–S5. Regarding DNA data analysis, from the total number of reads 2,741,939,082, 93% of the reads (2,550,198,134) were mapped, after trimming and alignment, with a global average quality (ratio between sum of bases qualities and total length) of 37.05 and 81.3% of reads were properly paired out (Supplemental Table S6). After trimming and alignment of DNA sequences of each sample a total 17,282,868 genetic variants were obtained with a total of 560,417 small insertions, 519,025 deletions (InDels) and a total 16,203,426 SNPs were detected. SNPs were filtered to remove SNPs with two (or three) possible alternative alleles in the Variant Call Format (VCF) file, obtaining a total of 15,692,912 SNPs. In this set, 70.80% were transitions (11,111,319 loci) and 29.2% transversions (4,581,593 loci), and the ratio of transition/transversion was 2.43 (Supplemental Table S7). Additional filtering to remove sites with missing data and also homozygous sites were performed using a custom script in python. As a result, 12,198,767 SNPs were retained for red cultivars and 11,128,404 SNPs for white cultivars. For the general analysis (using all samples) a total of 11,895,933 SNPs were retained. The results obtained by PopLDdecay showed that LD decays to 0.2 around 2 kb and to 0.1 around 300 kb (Fig. S1).

The analysis of the raw RNA data was performed for all 10 cultivars and all four stages (P, PV, EV and H) considered in this study. In the alignment step, a total of 1,692,026,464 sequences of red cultivars were aligned against the reference genome of the cultivar PN40024 (GCA_000003745.2)^28^ and a total of 1,702,609,789 were aligned in the case of white cultivars. After that, the RNA-Seq differential expression analysis was carried to compare only the two target stages, PV and EV, according to the previous results found by Massonnet et al.^27^. To assess data quality for outliers, in parallel with the differential expression analysis, the PCA function was used (Supplemental Fig. S2). In the case of PV and EV comparison for red cultivars, a total of 25,087 (59.14% of 42,416 total annotated genes in grape) genes with at least one count were detected. After that, 19,379 genes with summed counts greater than 20 were retained for differential expression analysis. After, the differential expression analysis 11,899 DEGs with an adjusted p-value cutoff (FDR) < 0.05 were detected. In this final set, the total number of DEGs with a positive log2foldchange (LFC) (up) was 5903 (30%) and with a negative LFC (down) was 5996 (31%).

For the target comparison (PV vs EV) in white cultivars, 25,470 (60.05% of the total annotated genes in grape) genes had at least one count and only 19,160 genes, with summed counts greater than 20, were retained for differential expression analysis. After the differential expression analysis 10,834 DEGs showed an adjusted p-value cutoff (FDR) < 0.05, with 5554 (29%) up and 5280 (28%) down. In the general study, 26,691 had at least one count, 20,621 showed more than 20 counts and were used for the expression analysis. Finally, 13,447 DEGs showed an adjusted p-value cutoff (FDR) < 0.05, with 6641 (32%) up and 6806 (33%) down regulated (Supplemental Table S8).

The obtained genotypic data and the expression data of the DEGs (up and down) identified was used as input for Matrix eQTL, to compare the selected stages (EV and PV). The number of *cis*-eQTL associations identified by MatrixEQTL was 2244 (2170 unique) (Supplemental Table S9). In the general eQTL analysis (using all genotypes together), 106 *cis*-eQTLs were identified by Matrix eQTL, associated with 22 down regulated genes (Fig. S3). In addition, 334 *cis*-eQTLs identified in the general analysis were associated with 36 up regulated genes (Fig. S3). For white cultivars, 867 *cis*-eQTLs were significant associated with 76 DEGs (Fig. S3). In the case of red cultivars, the total number of *cis*-eQTLs associations identified was 937 related with 105 DEGs (Table 1, Fig. S3). The intersection of genes between categories showed that white and red cultivars shared a high number of up regulated genes (11) and only one down regulated gene. Only two genes were shared between RU, WU and GU (Fig. S4).

**Table 1:** Number of significant *cis*-eQTLs associations and number of related DEGs identified by the eQTL analysis.

	Num. of *cis*-eQTLs	DEGs
General down (GD)	100	22
General up (GU)	313	36
White cultivars	867	76 (12 down and 64 up)
Red cultivars	937	105 (29 down and 76 up)

**Figure 1:**
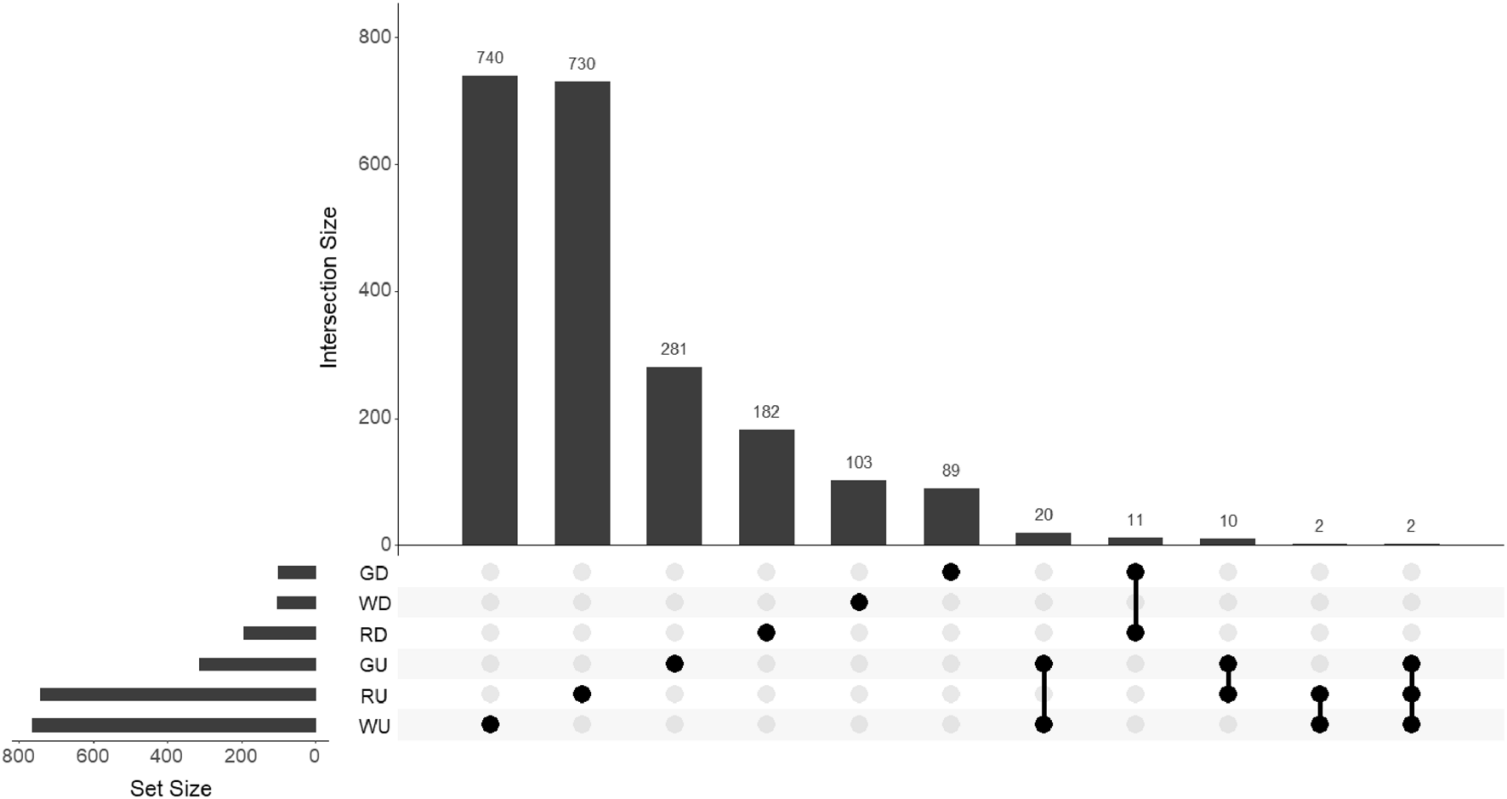
Intersections of the results from the eQTL analysis, showing unique and common *cis*-eQTLs in down regulated genes in white cultivars (WD), up regulated genes in white cultivars (WU), down regulated genes in red cultivars (RD), up regulated genes in red cultivars (RU), down regulated genes after the general analysis (GD) and up regulated genes after the general analysis (UD).

The intersection of the results showed that 89 *cis*-eQTLs were unique for GD and 281 for GU. A total of 912 *cis*-eQTLs were unique for red cultivars and 843 *cis*-eQTLs unique for white cultivars (Fig. 1). Only two *cis*-eQTLs were shared uniquely between read and white cultivars (NC_012020.3_22792433_4612345, NC_012021.3_2416735_4947832) associated with two genes Vitvi14g02910 and Vitvi15g00121 both up regulated in PV stage.

For each DEG, the number of *cis*-eQTL associations was variable ranged from one *cis*-eQTL (48 DEGs) to 142 *cis*-eQTLs (Vitvi17g00576). Other DEGs with large number of *cis*-eQTL associations were Vitvi11g00498 (55), Vitvi03g01373 (65), Vitvi16g00122 (94). The complete information about gene ID and *cis*-eQTL can be found in (Supplemental Table S9).

**Figure 2:**
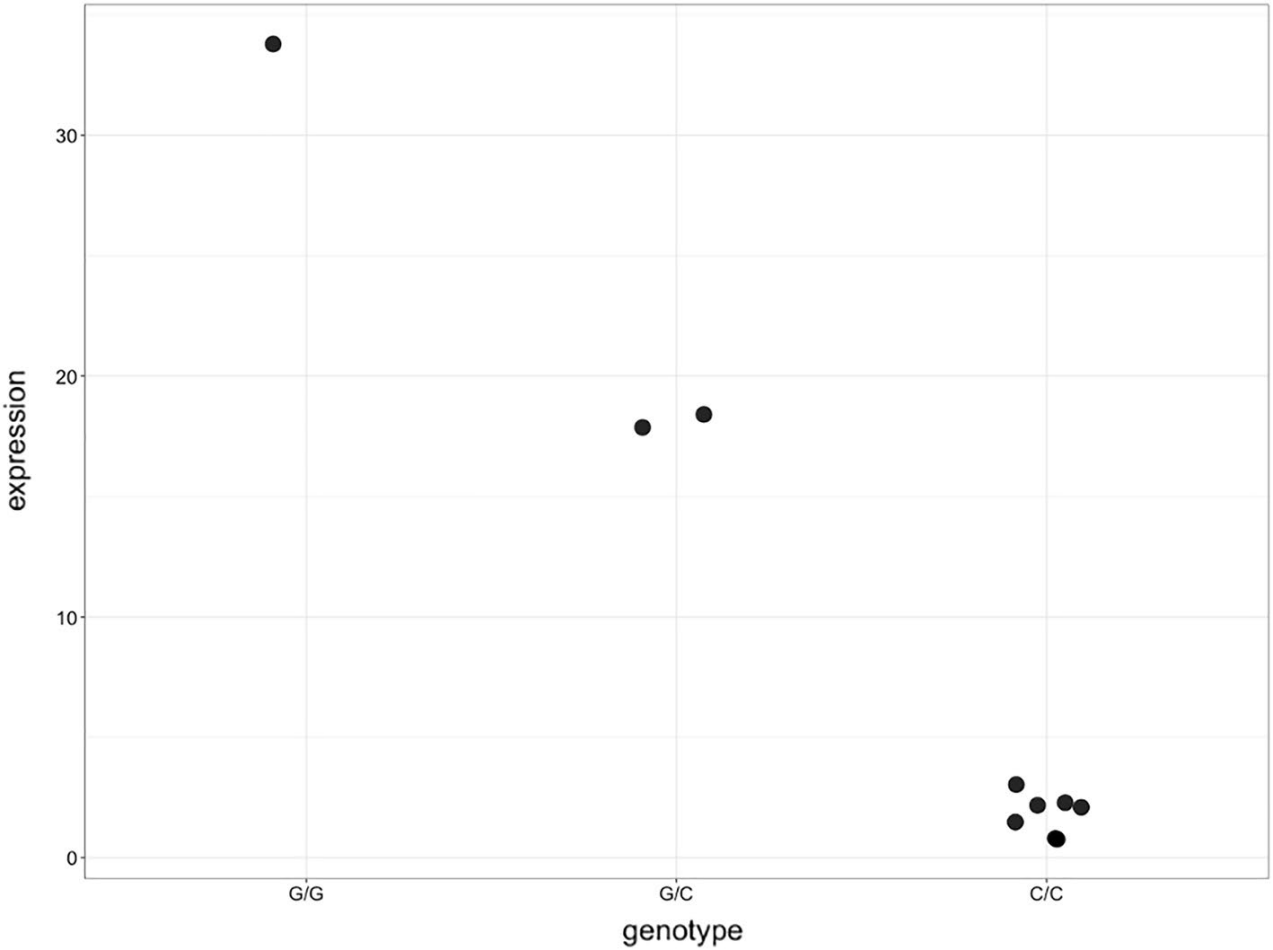
Differential expression of the different genotype classes detected for the *cis-*eQTL NC_012015.3_22440993_17257909 associated with Vitvi09g02012.

All the detected *cis*-eQTL associations, in the case of red cultivars and white cultivars, separately, showed only two genotype classes [homozygous (AA) and heterozygous (AB) or homozygous (BB) and heterozygous (AB)], and the homozygous state for the second allele was not observed. Only in the general comparison of down and regulated genes, with all the cultivars together, 72 *cis*-eQTLs showed three genotype classes (AA, AB, BB) in down regulated genes and only five *cis*-eQTLs showed three classes in up regulated genes (Supplemental Table S10). These 77 *cis*-eQTLs were associated with four DEGs (Vitvi12g02656, Vitvi03g01373, Vitvi09g01367, Vitvi09g02012) (Supplemental Table S9). In the case of Vitvi09g02012, its *cis*-eQTL associated (NC_012015.3_22440993_17257909) represents a change from GG (mean value of expression for GG genotypes is 1.81) to CC (expression of CC genotype 33.70), which represents more than 173% of increment of the gene expression. The GC genotypes have an intermediate expression value of 18.01 (Fig. 2).

The effects of the *cis*-eQTLs identified on their related DEGs (212 down and up regulated genes) were 3065 according to SnpEff (Supplemental Table S11). The 1.83% (56) of these effects were classified as high (with disruptive impact on the protein), 4.08% (125 effects) were low effect (with synonymous substitution), 7.83% (240) were moderate (genetic variation could have a non-synonymous substitution) and 86.26% (2644) were modifier effects (with impact on noncoding regions). The most frequent mutations with high impact effects were stop gain variants and frameshift variants (39.29% and 23.21%, respectively). The majority of the total effects (3065) were located in introns (806 effects, 26.29%), downstream (627 effects, 20.45%) and upstream regions (625 effects, 20.39%).

The 56 high impact *cis*-eQTLs identified in this study were related with 32 DEGs (Supplemental Table S11). In the majority of these genes at least one high impact *cis*-eQTL association was observed, five DEGs showed two high impact *cis* associations (Vitvi12g01443, Vitvi16g01175, Vitvi18g00307, Vitvi06g00354, Vitvi07g02574 and Vitvi07g00061), two DEGs showed three *cis* associations (Vitvi04g02151 and Vitvi12g02623), three DEGs showed four high impact associations (Vitvi16g00122, Vitvi12g01425, Vitvi06g00918) and one DEGs showed six high impact *cis* associations(Vitvi07g00414).

The functional characterization of each DEG, showed 80.66% (171), received a UniProtKB^29^ identifier, and 14 (6.60%) received a DAVID^30^ term according to the annotation analysis (Supplemental Table S12). Finally, Gene Ontology (GO) showed that DEGs were associated with terms as defence response (GO:0006952), response to light stimulus (GO:0009416), response to auxin (GO:0009733), response to gibberellin (GO:0009739), response to abscisic acid (GO:0009737) and cellular response to salicylic acid stimulus (GO:0071446) (Supplemental Table S13). Genes associated with these GO terms coding for protein such as growth-regulating factor, MADS-box domain-containing protein, RING-type domain-containing protein, PHD finger protein ING, Phytocyanin domain-containing protein, Kinesin motor domain-containing protein, Xyloglucan endotransglucosylase/hydrolase (EC 2.4.1.207), DLH domain-containing protein, ANK_REP_REGION domain-containing protein (Supplemental Table S12).

## Discussion

A detailed bioinformatic pipeline has been established to study an important phenomenon such as fruit ripening in an important woody crop species such as *Vitis vinifera* L. The information about genetics of gene expression obtained in this study provides researchers with new scenarios to understand this genetically programmed process that is physiologically and biochemically irreversible. The two main criticisms of this study can be the low number of genotypes used and that a high percentage (48.11%) of the 212 DEGs code for an uncharacterized protein. Both issues could be explained by the difficulty of working with woody crops and because *Vitis vinifera* is not a real model species in plants like *Arabidopsis thaliana*. As commented by Mudge and Harrow^31^, in higher eukaryotes gene annotation projects are highly complex reflecting the complexity that survives in eukaryotic cells, and more important at the present time all of our genebuilds (representations of the transcriptome that exists in nature) are incomplete.

Our results support rapid decay of LD for this species. According to literature, thousands of years of widespread vegetative propagation has resulted in a weak domestication bottleneck in grapes^32,33^. The fast decay LD observed here could suggest that the genes detected could be regulated by different SNPs independently. In fact, different values of LD were observed across four genomic regions between wine eastern cultivars, wine western varieties, eastern table grapes and wild grapevine individuals by Nicolas et al.^34^. A fine mapping approach, using software as FINEMAP^35^, or TreeMap^36^, could be necessary to detect the lead eQTL signals on specific regions (and genes). Fine mapping has been carried out in plants such as maize to understand one of the key steps in its domestication^37^.

The molecular and biochemical phases leading to the initiation of ripening harbor a level of complexity not fully understood^38^. The results reported by Fortes et al.^38^, identified several functional categories such as "development", "metabolism", "diverse/miscellanenous functions'', "cellular process", "regulation overview" or "response to stimulus, stress" together with several not previously identified genes in the context of grape ripening. The same authors observed that abiotic (e.g. osmotic, temperature) stress responses and biotic (e.g. pathogens) increase during grape ripening^38^. In the present study, these functional categories detected by Fortes et al.^38^ such as "response to stimulus, stress" or “developmental process” were also identified. In this sense, our results have provided a new list of candidate genes that can help to understand grape ripening better, something that is still far from being completed.

With the GO analysis obtained here, clear differences can be observed between the two stages of growth studied, a large set of high expressed genes in PV stage (and down regulated in EV) were annotated as with terms as defense response, response to light stimulus, response to auxin, response to gibberellin and response to stress. Up regulated genes in EV stage (and down regulated in PV) were annotated to response to oxidative stress or response to abscisic acid. In this sense, exogenous stimuli (temperature, light, abscisic acid, jasmonic acid and oxidative stress) have a lower impact in véraison-stage berries, which seem to have a greater resilience to metabolic alteration driven by these factors, than pre-véraison stage berries^39^. According to the literature, the accumulation of polyphenols in early stages seems to offer a strategy for the defense of the ripening berry^40^; otherwise, the stress input is overcome by the developmentally regulated metabolic program. More interestingly, a large amount of gene IDs identified by Matrix eQTL have not previously been identified in the context of grape ripening. The obtained characterization of regulatory variants can now provide a valuable resource to help the biological understanding of grape ripening.

According to the literature, after the véraison stage, the content of several hormones, as auxins and cytokinins, decrease while abscisic acid concentration increases^41^. Auxins are phytohormones associated with an extensive variety of phases of the development. In addition, to the classical link with growth of these phytohormones at early stages of fruit development, others functions, such as the ability to affect ripening, have been inferred^42^. In the present study, the gene Vitvi11g00497 gene is upregulated (2.52 log2FC) in PV stage in comparison with EV, coding for auxin-responsive protein, it could suggest a possible role of this gene as a repressor of early auxin response genes at low auxin concentrations.

Germin-like proteins (GLPs) are expressed in several developmental phases and organs in plants, as a consequence of a number of abiotic and biotic stresses^43^. In a fruit species such as plum (*Prunus salicina*), two novel GLP genes were isolated, exhibiting similar expression patterns throughout several phases of fruit development, except pit hardening and fruit ripening. According to the results, the accumulation of both Ps-GLP*s* is related to the evolution of auxin, since fruit develop until the ripening stage, suggesting that auxin is affecting the regulation of both transcripts throughout the development and ripening of the fruits^44^. The Vitvi14g02553 gene, down regulated (− 6.34 log2FC) in PV, was associated with four *cis*-eQTLs by Matrix eQTL in red cultivars. The low regulation in an early stage of fruit development (pre-véraison) could suggest that this gene is putatively accumulated in the fruit during ripening in an auxin dependent manner.

Another interesting gene was Vitvi11g01682 up regulated in PV stage, this gene is coding for a Xyloglucan endotransglucosylase/hydrolase (EC 2.4.1.207) protein. The gene was associated with six *cis*-eQTLs, one of them (NC_012017.3_19311933_1553582) was annotated by snpEff as frameshift_variant, having a high impact, because the number of nucleotides inserted or deleted is not a multiple of three. The Xyloglucan endotransglucosylase/hydrolase (XTHs: EC 2.4.1.207 and/or EC 3.2.1.151), a xyloglucan modifying enzyme, seems to play an important role in fruit ripening. Different XTHs seem to be subjected to a different expression pattern regulation during fruit growth and ripening^45^, in our study Vitvi11g01682 was up regulated in PV stage. XTH genes have been related to fruit growth and ripening in different climacteric fruits such as tomato, apple, kiwi fruit, pear, persimmon or cherimoya fruits^45^. Two XTH genes (SIXTH and SIXTH9) showed a dramatic reduction in mRNA levels immediately prior to the onset of ripening, around the mature green (MG) stage in tomato^46^. Our results have confirmed an early pattern of expression for this gene, which could support the role of this gene in wall modification in grape.

Another identified genes, all of them up regulated in PV with and with *cis*-eQTL associations detected in red cultivars, were Vitvi12g02292, Vitvi18g00307 and Vitvi08g01770. These genes were annotated such as ROMT (Resveratrol *O*-methyltransferase), flavonol synthase (FLS) and terpene synthase, respectively, by the Integrape Catalogue 2.3^47^. FLS is a key enzyme in flavonol biosynthesis that control the last step, from dihydroflavonol to flavonol^48^. According to the literature, five FLS genes are present in the grapevine genome^53^. According to Fujita et al.^49^, the mRNA of FLS2, accumulated in small berry skins and then decreased toward véraison, in our study FLS2 [Vitvi18g00307 (VIT_18s0001g03510)] was up regulated in PV (pre-véraison). This gene showed 18 *cis*-eQTLs associations in red cultivars, two of them had a high impact effect (a stop_lost and frameshit_variant and stop_gained). Previous results have established that VvMYBF1 (accession FJ948477), located in chromosome 7 of cultivar PN40024 and with an expression extremely sensitive to light induction^50^, is a specific activator of flavonol synthase1 [VvFLS1 (Vitvi18g02542)]. In our study two upregulated DEGs (Vitvi11g00498, Vitvi09g01008) in PV and one down regulated DEG in PV (Vitvi08g00654) were annotated as HTH myb-type domain-containing protein and as partial protein by NCBI (accessions: CBI28231.3, CBI38956.3, CBI22798.3, respectively). These results could suggest a possible role of some of these genes in the activation of FL2, which should be further investigated in grape. A summary of the information obtained in this study for each candidate gene mentioned above can be found in Supplemental Table S14.

This study has provided new insights in grape ripening and manifests the advance of integrating different omics data for the study of complex processes and traits. The accessibility to data underlying scientific publications allowed the author to efficiently apply this exhaustive methodology. Clearly, this study has exploited the value of mandated public data archiving (PDA) policies in the sciences, which is mandatory for top journals in several areas, including evolution and ecology. Overall, this work will improve the understanding of gene regulation during fruit ripening from pre-véraison to post-véraison in *Vitis vinifera* L. Also, this work detected *cis*-eQTLs effects of big size, as in the case of NC_012015.3_22440993_17257909 associated with Vitvi09g02012, which disagree with the results of large-scale experimental reports of putative regulatory variants^6^ where most eQTL effects are of relatively small size (< 2-fold change in expression)^14,51^. In the future, in addition of the detection of the lead eQTLs, the detected genes could be used for functional and mechanistic studies of grape ripening. Finally, and more importantly, the approach developed here could be applied to other woody crops species, such as fruit trees or nut trees, where these types of studies are scarce and when the availability of such comprehensive data sets becomes a reality.

## Materials and methods

### Plant material

DNA and RNA data from five red-skinned grape cultivars (“Barbera nera”, “Sangiovese”, “Refosco”, “Negro amaro” and “Primitivo”) and five white-skinned grape cultivars (“Garganega”, “Vermentino”, “Moscato bianco”, “Glera”, and “Passerina”) grown at the CREA-VE grapevine germplasm collection (Susegana, Veneto region, Italy) were used in the current study. Four different growth stages were considered for each variety: pea-sized berries (Bbch 75) at almost 20 days after flowering (P), berries beginning to touch (Bbch77) just prior véraison (PV), the softening of the berries (Bbch 85) at the end of véraison (EV) and berries ripe for harvest (Bbch 89) (H). A more detailed description about berries collection can be found in^27^.

### DNA-seq and RNA-seq data

**Figure 3:**
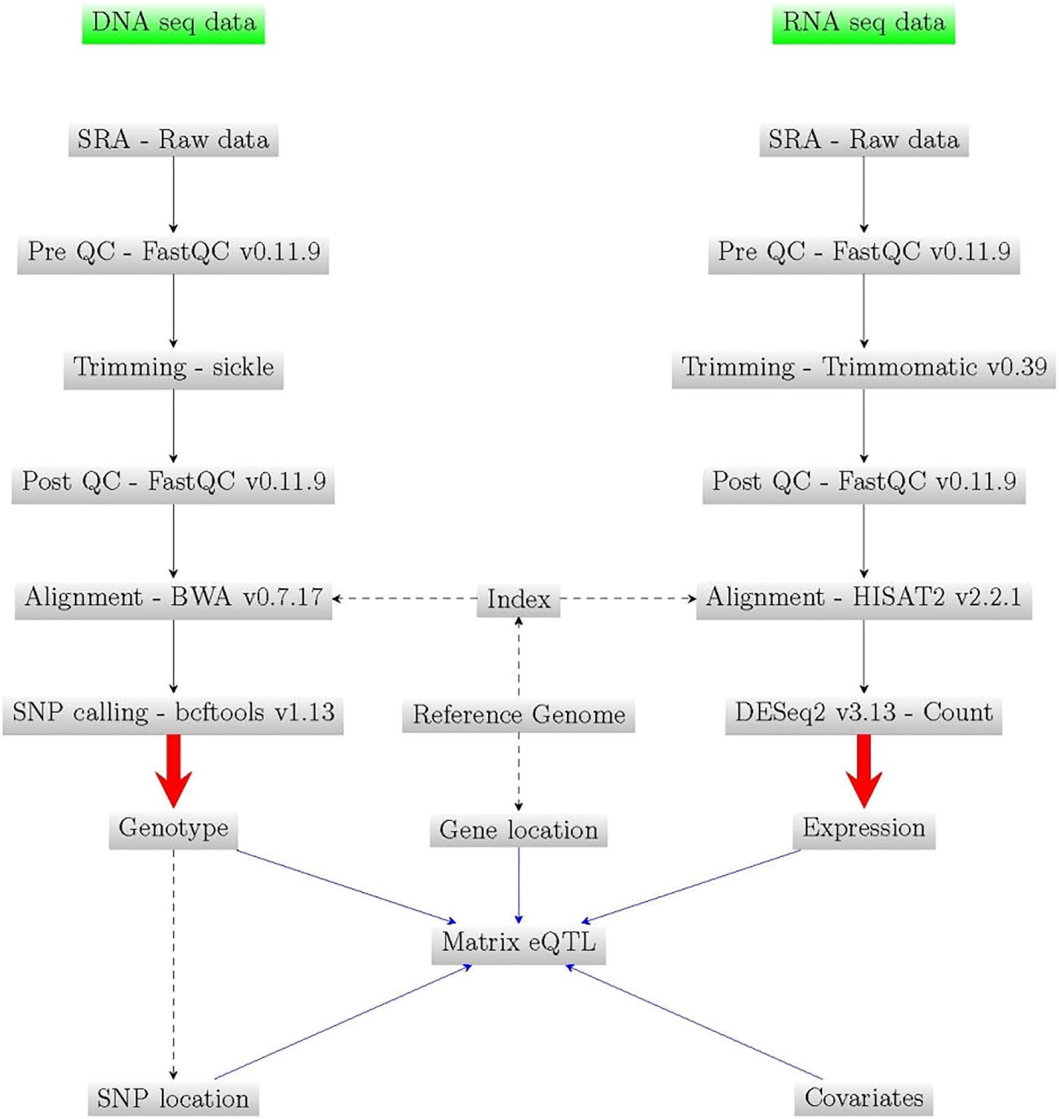
General workflow of the study. The workflow was drawn using the LaTeX package TikZ^53^.

Both set of data were collected from National Center for Biotechnology Information (NCBI) Short Read Archive (SRA, https://www.ncbi.nlm.nih.gov/sra) obtained from previous studies^21,52^. All sets of sequences were downloaded with the SRA Toolkit v2.11.1. The main difference regarding DNA data processing with the previously published study of these cultivars is the use of a different pipeline for SNP calling. Annotated genes in grape were obtained from Vitis_vinifera_gene_an notation_on_V2_20.gff3. The complete workflow generated here can be observed in Fig. 3.

### DNA-seq data analysis

The raw data were obtained from three BioProject PRJNA385116^52^, PRJNA392287 and PRJNA373967^21^. Briefly, in order to evaluate the general quality of reads in each file the FastQC v0.11.9 was used. The command used can take multiple files as input and outputs a quality report in html format. After that, low quality bases and/or adapter contamination were removed from reads using the techniques in Joshi and Fass^54^. This step would discard any read trimmed shorter than 45 bp. FastQC was run again on the trimmed data to confirm and ensure the final quality of reads. After this second quality control (QC) step, and before the alignment step, the reference genome was indexed using the command index of BWA v0.7.17^55^. Then, the V. vinifera 12Xv2 reference genome^28^ of the strain PN40024 (GCA_000003745.2) was used to align the reads using BWA v0.7.17^55^. BWA is one of the most widely used short-read aligners. BWA implements several alignment methods, but mem was selected. Reads were aligned with the default parameters. The output from an aligner such as BWA are in Sequence Alignment/Map (SAM) format. After this process the SAM files were compressed to Binary Alignment Map (BAM) format using samtools v1.13^56^. Reads were sorted by their positions in the reference genome using picard tools^57^. A BAM index was created and the variant calling was carried out using the methods implemented in bcftools v1.13^56^. Finally, to remove low quality variants (e.g., variants with a low read depth or variants only supported by poorly aligned reads) from our data set, a quality filtering was applied to the VCF file using the command bcftools view (bcftools view -i 'QUAL > 19 && DP > 2$ && (AC/AN) > 0.05 && MQ > 20'). In our study, the VCF file was used for linkage disequilibrium (LD) decay analysis, using the software PopLDdecay v3.40^58^.

### RNA-seq data analysis

The complete information of the yielded 120 SRA files (10 varieties at four stages, in total 40 triplicate samples), downloaded from two BioProjects PRJNA265040^21^ and PRJNA265039^21,27^, can be found in Tables S2 and S3. Briefly, after the quality control (QC) step, Trimmomatic v0.39^59^ was used to trim low quality and adapter contaminated sequences. In this case, the alignment of reads to the reference genome was performed by HISAT2 v2.2.1^60^. HISAT2 is a fast and sensitive aligner for mapping next generation sequencing reads against a reference genome. Before the alignment, the hisat2 build module was used to make a HISAT index file for the genome. By default, HISAT2 outputs the alignments in SAM format. Samtools was used to sort the sequences, convert them to binary format and compress them. Finally, the function htseq-count from the HTSeq v0.13.5 package^61^ was used to count how many reads map to each annotated exon (gene) in the genome. The final count for each gene was obtained from sum values for all their exons. These final counts per gene are the inputs of the R package DESeq2 v3.13^62^, used for the differential expression analysis.

A complete tutorial, to reproduce the reported results has been generated. All scripts and datasets used and generated during the current study are available in the GitHub repository https://github.com/pjmartinez/TFM_UM-eqtls.

### Matrix eQTL

Matrix eQTL v2.3^63^ was used for the eQTL analysis. This software can accommodate large expression and genotype datasets. Matrix eQTL checks for association between each SNP and each transcript by modeling the effect of genotype as either categorical (ANOVA model) or additive linear (least squares model). The simple linear regression (used in this study) is one of the most commonly used models for eQTL analysis. In addition, Matrix eQTL can test for the significance of genotype-covariate interaction (not considered in this study). Matrix eQTL also supports correlated errors to account for relatedness of the samples and heteroscedastic. Matrix eQTL implements a separate test for each gene-SNP pair and corrects for multiple comparisons by calculating false discovery rate (FDR)^64^. Five different input files (snps = snps data; gene = mean by sample of the normalized read counts of each DEG; cvrt = covariates; genepos = gene location; snpspos = SNP location) are required to run Matrix eQTL. All these files need to have a specific format. The columns of all three files must have matching order, corresponding in each column to a sample and with one gene/SNP/covariate in each row. In the case of the genotype file, if a linear model is used, as in this study, the values must be numerical in this data set. For that reason, extract.gt function from the R package vcfR v1.12^65^ was used to read and extract genotypes from our VCF filtered file in numeric format. The p-value threshold for *cis*-eQTLs (pvOutputThreshold.cis) in this study was 1e−8 and the maximum distance at which gene-SNP pair is considered local (cisDist) was 1000. In our study, covariates were not considered. The location of each gene was obtained from the gff3 file: Vitis_vinifera_gene_annotation_on_V2_20.gff3 available at https://urgi.versailles.inra.fr/Species/Vitis/Annotations. The location of each SNP was obtained from the VCF file obtained.

To find *cis*-eQTL associations with down and up regulated genes [in red and white cultivars and in general (without consider colour of cultivars)], Matrix eQTL was run six times:

1. To detect *cis* eQTLs in down regulated genes in white cultivars (high expression in EV).
2. To detect *cis* eQTLs in up regulated genes in white cultivars (high expression in PV).
3. To detect *cis* eQTLs in down regulated genes in red cultivars (high expression in EV).
4. To detect *cis* eQTLs in up regulated genes in red cultivars (high expression in PV).
5. A general analysis to detect *cis* eQTLs in down regulated genes (high expression in EV).
6. A general analysis to detect *cis* eQTLs in up regulated genes (high expression in PV).

As a result of each run, each significant gene-SNP association was recorded in a separate line in the output file. Every record contains a SNP name, a transcript name, estimate of the effect size, t or F-statistic, p-value, and FDR.

After Matrix eQTL, the intersection(s) of common *cis*-eQTLs identified by Matrix eQTL and common genes between categories were detected using the R package UpSetR^66^. It is a quick approach to indicate which elements are in each intersection or are unique to certain conditions.

Prediction of genetics variants effects was performed using SnpEff v4.3e^67^ based on the grape gene annotation (Vitis_vinifera_gene_annotation_on_V2_20.gff3; https://urgi.versailles.inra.fr/Species/Vitis/Annotations). The SNP predicted effects were categorized by impact, as moderate (non-synonymous substitution); modifier (with impact on noncoding regions); low (synonymous substitution); or high (disruptive impact on the protein).

## Supplementary Information

Supplementary Tables.

Supplementary Figures.
