# Supplementary material for: Bioinformatic approach for the discovery of cis-eQTL signals during fruit ripening of a woody species as grape (Vitis vinifera L.)
Source: Sci Rep. 2022 May 6;12:7481. doi: 10.1038/s41598-022-11689-5 (PMC9076688; doi:10.1038/s41598-022-11689-5)

**Supplementary Figures**

Figure S1. A Scatter plot of LD decay (r2) against the genetic distance (300 Kb) for pairs of linked variants across all chromosomes and scaffolds in the VCF. B Zoom-in scatter plot of LD decay (r2) against the genetic distance (10 Kb).


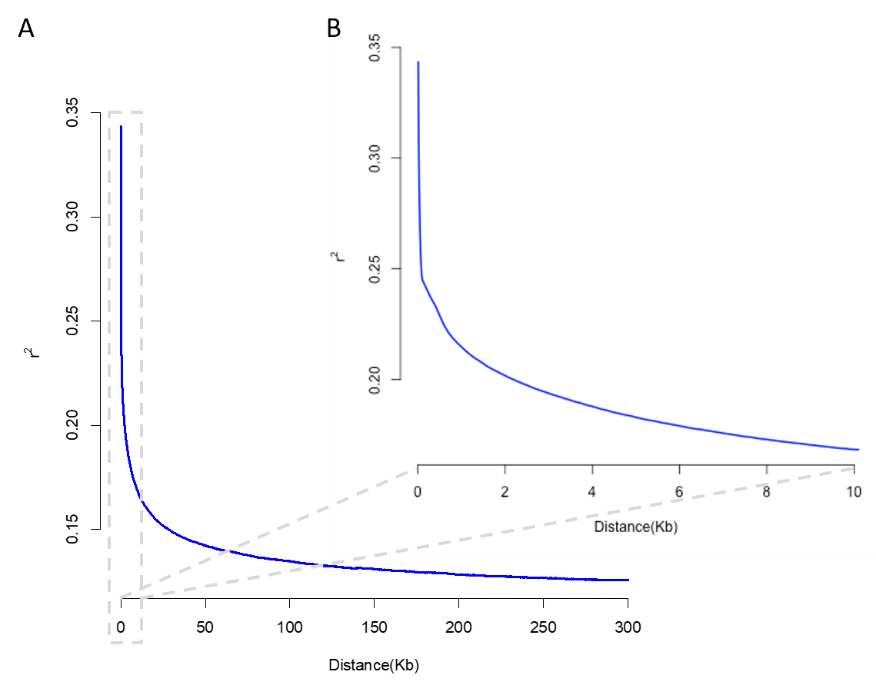


Figure S2. Principal Component Analysis between EV (red) and PV (blue) conditions. A) Red grapes B) White grapes c) General analysis. Each dot represents a sample.


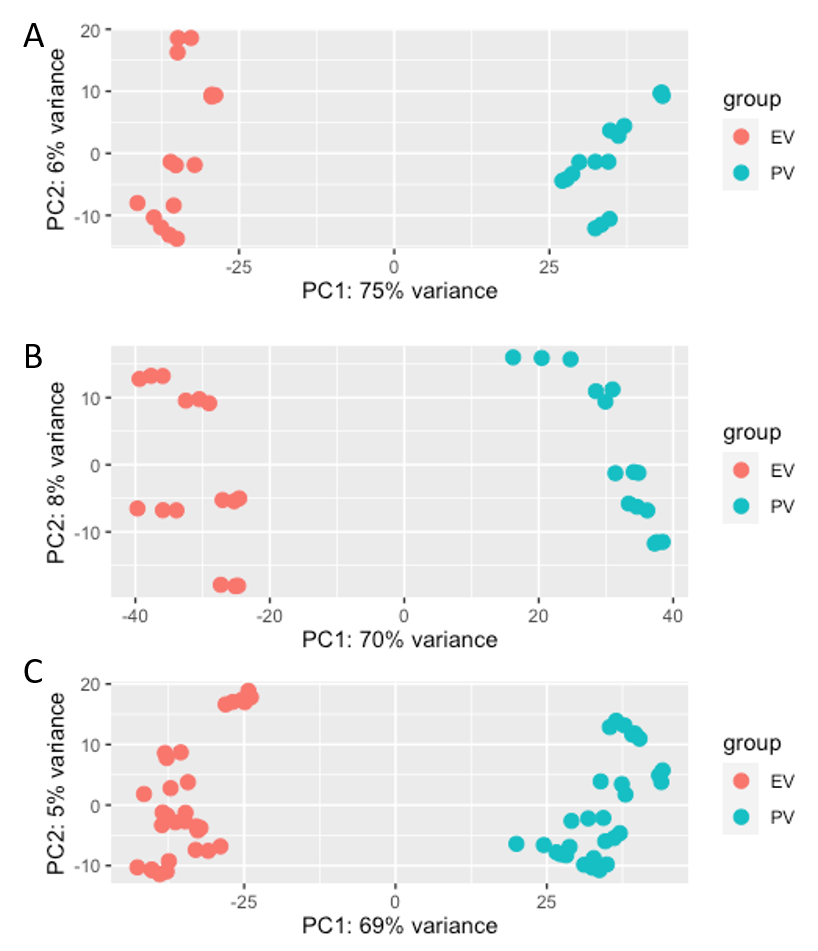


Figure S3. Differential expressed genes for each category. A) Down and up regulated genes between EV and PV (58 DEGs). B) DEGs between EV and PV in white cultivars (76 DEGs). C)DEGs between EV and PV in red cultivars (105 DEGs).


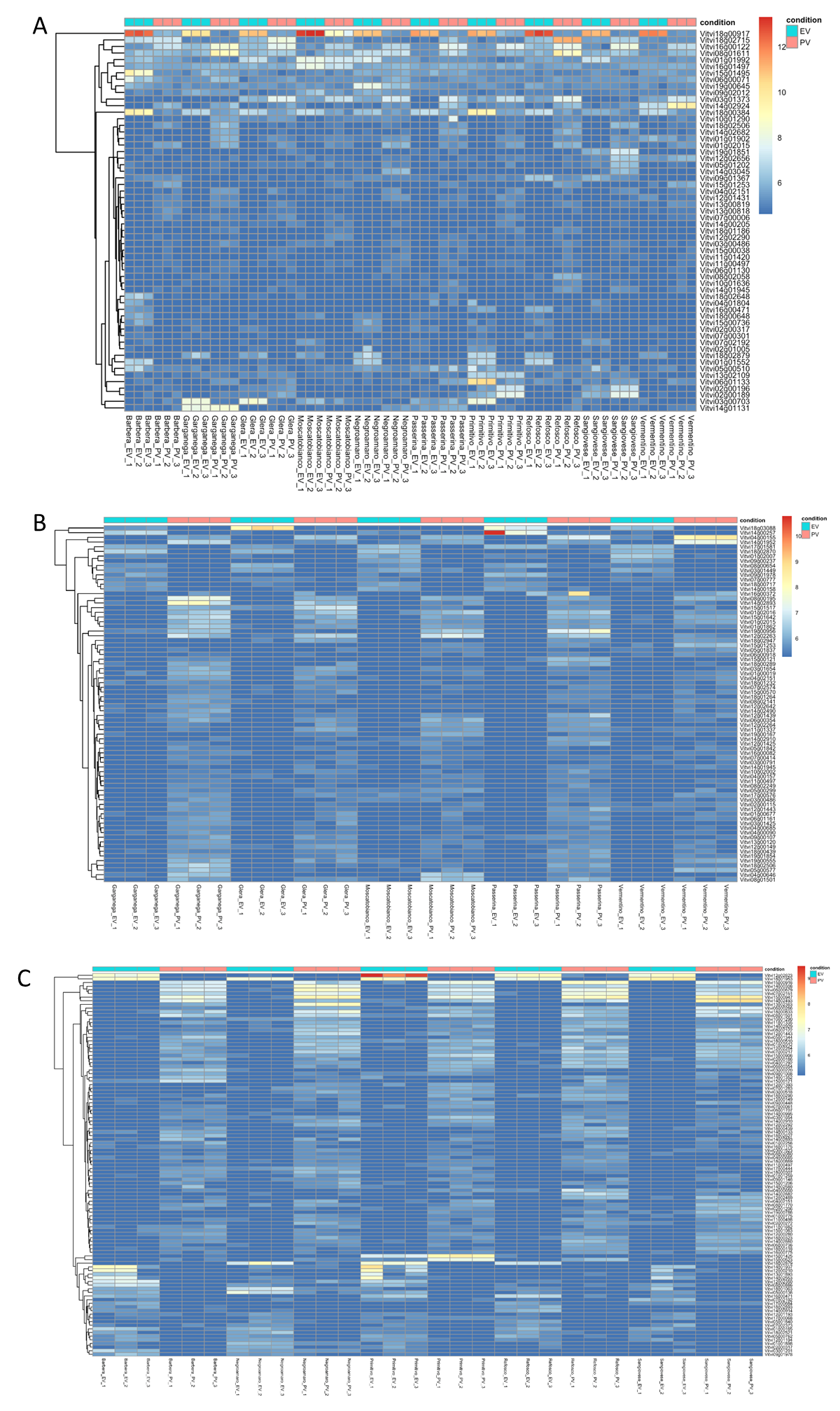


Figure S4. Intersections of DEGs between down regulated genes in white cultivars (WD), up regulated genes in white cultivars (WU), down regulated genes in red cultivars (RD), up regulated genes in red cultivars (RU), down regulated genes after the general analysis (GD) and up regulated genes after the general analysis (GU).


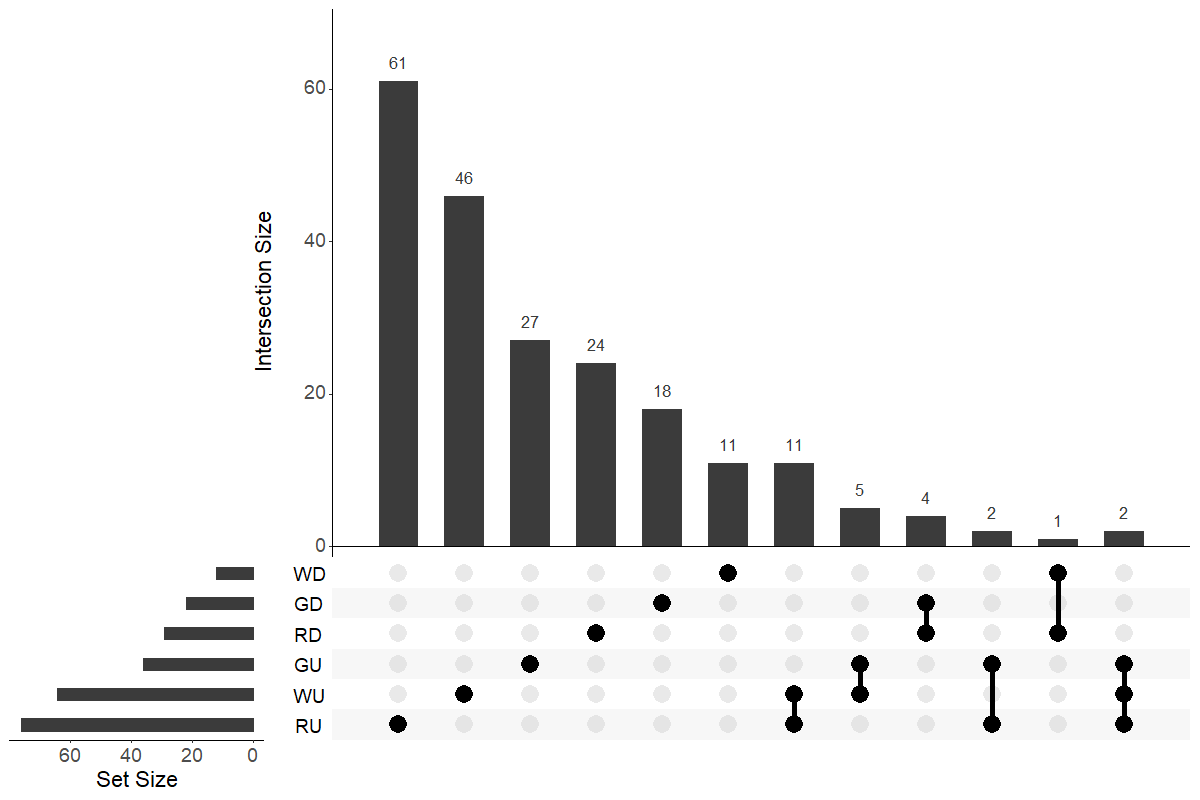

Supplement: Supplementary file 2 — Supplementary Figures. [file 41598_2022_11689_MOESM2_ESM.docx]
